# Aberrant *Ki‐67* expression through 3′UTR alternative polyadenylation in breast cancers

**DOI:** 10.1002/2211-5463.12364

**Published:** 2018-01-26

**Authors:** Hong Yan, Rui Tian, Wei Wang, Min Zhang, Jing Wu, Jie He

**Affiliations:** ^1^ Department of Pathology Anhui Provincial Hospital affiliated to Anhui Medical University and Anhui Provincial Cancer Hospital Hefei China; ^2^ Department of Medical Oncology Anhui Provincial Hospital affiliated to Anhui Medical University Hefei China

**Keywords:** 3′UTR, alternative polyadenylation, breast cancer, Ki‐67, miR‐140‐3p

## Abstract

Ki‐67 (MKI67) is a marker of cellular proliferation of cancer. Here, we show that Ki‐67 is post‐transcriptionally regulated through alternative polyadenylation (APA) and microRNAs in breast cancer. We show that shortening of the *Ki‐67* 3′UTR results in the loss of the binding sites for the suppressive miRNAs and thus renders the transcript with a shortened 3′UTR insusceptible to miRNA‐mediated suppression. This APA‐mediated shortening of the *Ki‐67* 3′UTR contributes to increased mRNA stability and enhanced translational efficiency. In summary, our results not only highlight the post‐transcriptional regulation of Ki‐67 involving APA and microRNAs but also suggest that *Ki‐67* 3′UTR disruption could serve as a molecular marker in breast cancer.

**Abbreviations**
3′RACE: 3′ rapid amplification of cDNA ends
APA: alternative polyadenylation
IHC: immunohistochemistry
PASs: polyadenylation signals

Breast cancer is the most common malignancy in women 1, 2. However, the molecular mechanisms of breast cancer are unclear. Prognostic factors, such as tumor size, nodal status, and histological grade, are the clinicopathological variables associated with the final outcome that are used to estimate the risk of death in breast cancer 3, 4.

Ki‐67 (MKI67) is a marker of cellular proliferation 5 and can predict the prognosis of patients with cancer 6, including breast cancer 7. In breast cancer, Ki‐67 is an established prognostic and predictive biomarker 8, 9, 10. The International Ki‐67 in Breast Cancer Working Group set standards for the staining, scoring, and analysis of Ki‐67 in breast cancer to ensure the reproducibility, reliability, and accuracy of studies using Ki‐67 as their primary measure of outcome 11. In addition, Ki‐67 is an independent prognostic factor in early breast cancer 9 and in neoadjuvant therapy 12, 13. High expression of Ki‐67 is associated with a poor prognosis in breast cancer 14. However, the mechanisms regulating Ki‐67 expression are largely unknown.

Alternative polyadenylation (APA) is a widespread phenomenon in the human genome 15, 16, 17. Over half of the human genes have APA signals 18. The importance of APA in human diseases, including cancer, has been emphasized by recent studies 19. The stability and translation efficiency of messenger RNA isoforms with shorter 3′UTRs, generated from APA, were increased due to the loss of miRNA‐mediated repression 20. Recently, studies have shown that the shortening of the 3′UTR may serve as a prognostic marker in cancer 21. Transcriptome‐wide studies have indicated a widespread shortening of the 3′UTR, which was frequently observed in various cancers, including glioblastoma, liver, lung, and breast cancer 22, 23, 24.

In the present study, we carried out 3′ rapid amplification of cDNA ends (3′RACE) and qRT‐PCR experiments to demonstrate the existence of three 3′UTRs of varying length in *Ki‐67* transcripts. Our results showed that the percentage of *Ki‐67* mRNA isoforms with shorter 3′UTRs was higher in the breast cancer clinical tissues compared to the matched noncancerous breast clinical tissues. Reporter assays revealed that the translation efficiency of shorter transcripts was higher than that of the full‐length transcripts, partly through the evasion of miRNA suppression. Taken together, our data highlight a novel regulatory mechanism of Ki‐67 and may lead to a deeper understanding of the role of Ki‐67 in cancer.

## Materials and methods

### Cell culture and transfection

Human breast cancer cell lines (MCF7, T47D, MDA‐MB‐453, MDA‐MB‐468, BT549, and MDA‐MB‐231) were purchased from the ATCC (Rockville, MD, USA). All cells were cultured under the recommended conditions and maintained in a humidified incubator at 37 °C. The transfection of the cells was performed with Lipofectamine 3000 (Invitrogen, Grand Island, NY, USA), according to the manufacturer's instructions.

### Breast tissue specimens

Twenty consecutive surgical breast cancer specimens and the corresponding adjacent nontumorous breast samples were obtained from Chinese patients at the First Affiliated Hospital of Anhui Medical University between 2006 and 2008. All specimens were confirmed as breast cancer based on histopathological evaluations. Tumor and normal specimens were snap‐frozen in liquid nitrogen and stored at −80 °C immediately after resection. Patients who had undergone chemotherapy or radiation therapy before surgery were excluded. The protocol of this study was approved by the Ethics Committee of Anhui Medical University. The work undertaken conforms to the provisions of the Declaration of Helsinki. Informed consent was obtained from all patients.

### Immunohistochemistry (IHC)

Formalin‐fixed, paraffin‐embedded tissue was cut into 4 μm sections, deparaffinized in xylene, rehydrated through graded ethanol, quenched for endogenous peroxidase activity in 3% hydrogen peroxide, and processed for antigen retrieval by heating in 10 mm citrate buffer (pH 6.0) at 90–100 °C. Sections were incubated at 4 °C overnight with Ki‐67 (1 : 500; Santa Cruz Biotechnologies, Santa Cruz, CA, USA). Immunostaining was performed using the UltraSensitive S‐P Detection Kit (KIT‐9720; Maixin, Fuzhou, China), and then, the color was developed using a DAB kit (DAB‐0031; Maixin). Subsequently, the sections were counterstained with hematoxylin. Quantification of immunohistochemical stain intensity was performed as previously described 25.

### Quantitative real‐time RT‐PCR

RNA was transcribed using the TaqMan Reverse Transcriptase Kit (Applied Biosystems, Foster City, CA, USA), and the resulting cDNA was used for real‐time quantitative PCR (Applied Biosystems 7900) using SYBR green PCR master mix from SensiMix SYBR^®^ from Bioline (Taunton, MA, USA). The TaqMan probes and primers were purchased from Shenggong (Shanghai, China). Human GAPDH was used as an endogenous control.

### Luciferase reporter assay

Luciferase reporter assays were performed using the psiCHECK2‐Ki‐67‐3′UTR vector. The Ki‐67 short, medium, and long 3′UTRs were amplified and cloned downstream of *Renilla luciferase* in a psiCHECK2 vector (Promega, Madison, WI, USA). Cells were grown to approximately 60% confluence in 24‐well plates and transfected with 100 ng plasmid or empty plasmid using Lipofectamine 3000. Forty‐eight hours after transfection, cells were lysed and analyzed with the dual‐luciferase assay (Promega) according to the manufacturer's instructions. Renilla/firefly luciferase readouts from the constructs were normalized to those of empty psiCHECK2, which was set to 1. Three independent experiments were performed in triplicate.

### 3′RACE and quantitative assessment of APA

One microgram of total RNA was used to generate cDNA with Superscript III reverse transcriptase (Invitrogen) according to the manufacturer's instructions using 3′RACE‐RT as a primer. The PCR was performed with a Ki‐67‐specific forward primer and 3′RACE‐R as a reverse primer. Primer pairs are as follows:

3′RACE‐RT: 5′‐GCGAGCACAGAATTAATACGACTCACTATAGGTTTTTTTTTTTTT;
3′RACE‐R: 5′‐GCGAGCACAGAATTAATACGACT;
Ki‐67‐F: 5′‐AGCAAATCTGTGCAGAGAGTAAC.

### Statistical analysis

Student's *t*‐test (two‐tailed) was used to compare two experimental groups; differences were considered statistically significant at *P *<* *0.05.

## Results

### *Ki‐67* mRNA with a shorter 3′UTR is upregulated in breast cancer

**Figure 1:**
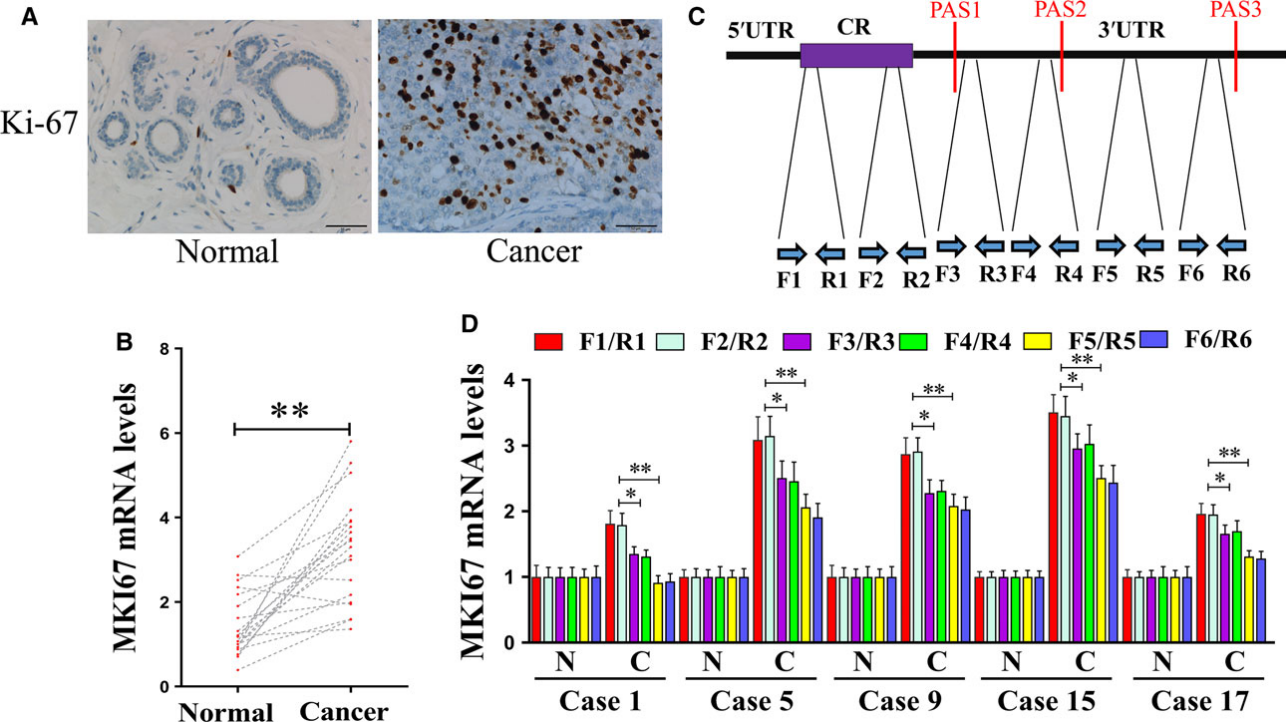
The expression of Ki‐67 protein and mRNA in breast cancer. (A) Immunohistochemical staining of Ki‐67 protein of breast cancer tissues and the corresponding noncancerous tissues. Strong immunostaining for Ki‐67 protein in breast cancer tissue and Ki‐67 protein expression is absent in noncancerous breast tissue. (B) The expression of *Ki‐67* mRNA is increased in breast cancer tissues compared to the corresponding noncancerous tissues. *Ki‐67* mRNAs were determined by RT‐qPCR, with *GAPDH* as the input control. (C) Schematic illustration of mRNAs with alternative isoforms due to APA. Positions of the PASs containing the AAUAAA or AAGAAA hexamer are indicated by vertical red lines. Positions of the primer pairs used in mRNA expression analyses are marked by black dashed lines. (D) Expression of *Ki‐67* mRNA isoforms containing various 3′UTR lengths evaluated by qRT‐PCR using primers specific for the long, medium, and short 3′UTRs. N‐adjacent normal cancer. C‐breast cancer. *P < 0.05; **P <* *0.01.

**Figure 2:**
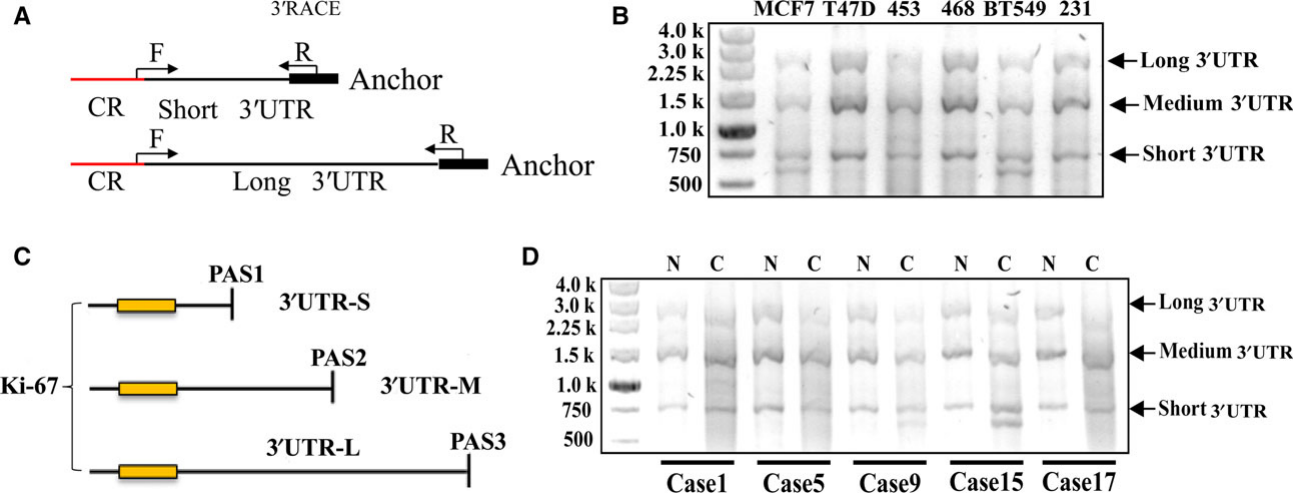
APA leads to shorter *Ki‐67* 3′UTR in breast cancer cells. (A) Schematic illustrations of the primer pairs used in 3′RACE analyses are marked by black arrows. Red lines show the protein coding region; black lines represent the untranslated regions. (B) Validation of the long or short *Ki‐67* 3′UTR transcripts by 3′RACE in breast cancer cells. (C) Schematic illustration of different *Ki‐67* mRNA isoforms with varying 3′UTR lengths mediated by APA. (D) Validation of the long or short *Ki‐67* 3′UTR transcript by 3′RACE in breast cancer tissue samples.

Initially, we compared Ki‐67 expression in breast cancer tissues and matched noncancerous breast tissues using IHC. The expression of Ki‐67 was significantly higher in all breast cancer tissues compared to the adjacent noncancerous breast tissues (Fig. 1A). Moreover, the expression of *Ki‐67* mRNA also increased in the breast cancer tissues compared to the adjacent noncancerous breast tissues (Fig. 1B). Recent studies have shown widespread shortening of the 3′UTR in cancer cells. We observed that the 3′UTR of the Ki‐67 gene contains three alternative polyadenylation signals (PASs; Fig. 1C). To determine the relative abundance of the *Ki‐67* mRNA isoforms terminated at the different PASs in the breast cancer cells, we performed RT‐qPCR using primers flanking different regions of the Ki‐67 3′UTR (F1/R1, F2/R2, F3/R3, F4/R4, F5/R5, and F6/R6; Fig. 1C). Interestingly, an increase in the short 3′UTR isoform of Ki‐67 was detected in five of the twenty breast cancer tissues compared to adjacent noncancerous breast tissues (Fig. 1D). Additionally, the 3′RACE assay was carried out to verify the relative abundance of the different *Ki‐67* mRNA isoforms (Fig. 2A). The *Ki‐67* mRNA isoforms terminated at PAS1 and PAS2 were designated 3′UTR‐short (3′UTR‐S) and 3′UTR‐medium (3′UTR‐M), respectively (Fig. 2B,C). In the 3′RACE assay, the percentage of the *Ki‐67* mRNA isoforms terminated at PAS1 (3′UTR‐S) and PAS2 (3′UTR‐M) were significantly higher in the breast cancer tissues compared to the adjacent noncancerous breast tissues (Fig. 2D).

### *Ki‐67* mRNAs with a shorter 3′UTR have greater stability and produce more protein

**Figure 3:**
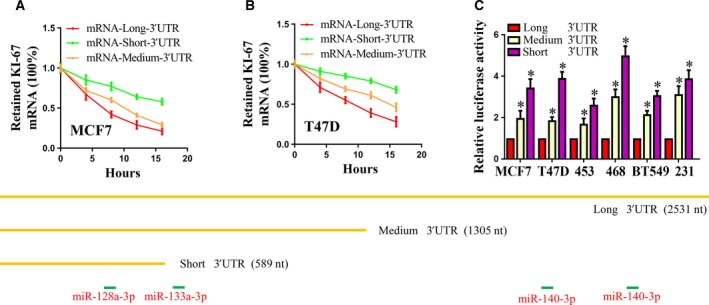
The shorter mRNA isoform leads to higher stability and protein expression than the full‐length isoform. (A) MCF7 and (B) T47D cells were treated with actinomycin D (10 μg·mL^−1^) 48 h after transfection and harvested at 0, 4, 8, 12, and 16 h for RNA extraction and reverse transcription. *Ki‐67* mRNA levels at the different time‐points were measured by qPCR, using *GAPDH* as the input control. (C) Luciferase reporter activities from a reporter containing the 3′UTR of the short or medium *Ki‐67* isoform, compared to that from the reporter containing the 3′UTR of the long *Ki‐67* isoform. The luciferase activity was measured by the dual‐luciferase reporter assay and is presented as Rluc/Fluc (renilla/luciferase firefly). The Rluc/Fluc value of the control was set as 1. (D) Graphical representation of the approximate position of the reported miRNA target sites are shown on long, medium, and short 3′UTRs. *P <* *0.05.

Recent studies have revealed that the production of mRNA isoforms with a shorter 3′UTR via APA results in increased mRNA stability and increased protein production. Thus, we examined the stabilities of the shorter and the longer *Ki‐67* mRNAs in the MCF7 and T47D cell lines. The total RNAs of the MCF7 and T47D cell lines were extracted after treatment with actinomycin D, and the expression of the mRNA was investigated by qRT‐PCR. The results showed that the shorter mRNA was more stable than the longer mRNA (Fig. 3A,B).

In addition to mRNA destabilization, to determine whether different isoforms produced different amounts of protein, we cloned each of the three 3′UTR isoforms of *Ki‐67* downstream of a luciferase reporter gene into the psiCHECK2 vector. The reporter mRNA isoform with the shortened *Ki‐67* 3′UTR exhibited a higher luciferase reporter activity compared to the *Ki‐67* 3′UTR‐medium and *Ki‐67* 3′UTR‐long reporter mRNA isoforms (Fig. 3C). These results suggest that the *Ki‐67* 3′UTR is shortened in breast cancer cells through APA, resulting in an increased *Ki‐67* translation efficiency.

### *Ki‐67* is targeted and differentially regulated by miR‐128‐3p, miR‐133‐3p, and miR‐140‐3p

**Figure 4:**
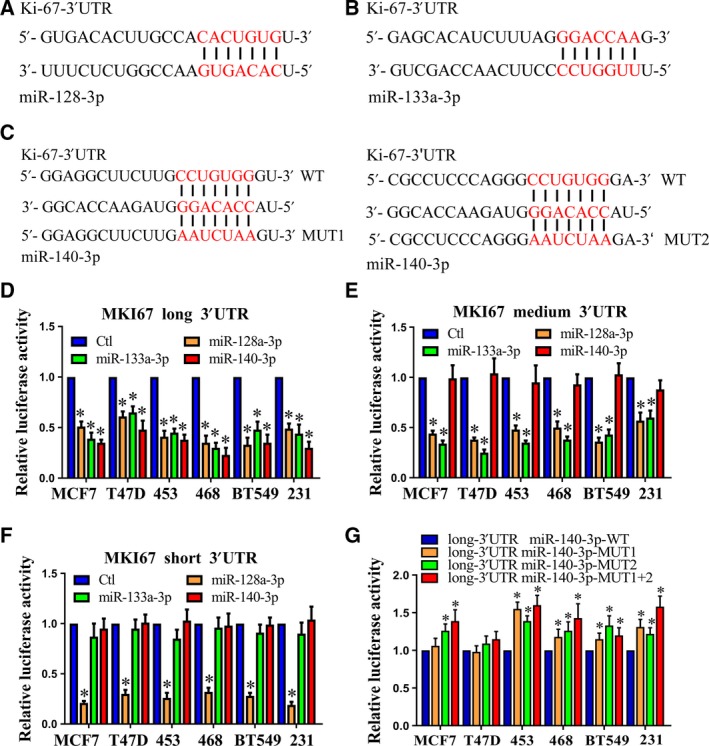
Contributions of miRNA regulation and 3′UTR length to the difference in luciferase activity observed between the long and short 3′UTRs. (A–C) The binding site of miRNAs within the *Ki‐67* 3′UTR. The region of the *Ki‐67* 3′UTR that interacted with miRNAs was identified by TargetScan and is highlighted in red. (D–F) Luciferase reporter activities were determined in MCF7 cells cotransfected with either control or different miRNA mimics, and either psiCHECK2‐Vec or psiCHECK2‐Ki‐67‐3′UTR, as indicated. (G) Luciferase expression of a reporter possessing the full‐length *Ki‐67* 3′UTR with mutant miR‐140‐3p sites is compared with that of a reporter with intact miR‐140‐3p sites in breast cancer cells. The luciferase activity was measured by the dual‐luciferase reporter assay and is presented as Rluc/Fluc (renilla/luciferase firefly). The Rluc/Fluc value of the control was set as 1. *P <* *0.05.

We next investigated potential miRNA target sites in the 3′UTR region of *Ki‐67*. Using TargetScan 26 and RNA22 27, we predicted numerous miRNA sites in the 3′UTR of the full‐length *Ki‐67* transcript, and most of these miRNA target sites, such as miR‐140‐3p and miR‐133a‐3p, were lost in the shortest isoform (Fig. 3D). Our luciferase reporter assays validated the functional importance of three of these predicted miRNAs (Fig. 4A–C).

After miR‐128‐3p mimics the transfection of MCF7 and T47D cells, a significant decrease in luciferase activity of all forms of 3′UTRs was observed (Fig. 4D–F). After miR‐133a‐3p mimics the transfection of MCF7 and T47D cells, a significant decrease in luciferase activity of the long and medium 3′UTRs was observed, while the short variant was not affected (Fig. 4D–F). Moreover, after miR‐140‐3p mimics the transfection of MCF7 and T47D cells, a significant decrease in luciferase activity of the long 3′UTRs was observed, while the short and medium variants were not affected. These data show that miR‐128‐3p, miR‐133‐3p, and miR‐140‐3p not only target *Ki‐67* but also target the position of the 3′UTR.

To determine whether the miRNAs contributed a greater translational repression for the longer 3′UTR, we mutated the miRNA complementary sites of miR‐140‐3p, downstream to the proximal polyA sites. The two miR‐140‐3p sites in the *Ki‐67* 3′UTR were mutated in the context of the long 3′UTR. The results showed that loss of the miRNA sites led to significant increases in luciferase activity in four breast cancer cell lines (Fig. 4G). However, the luciferase activity was slightly increased in T47D (Fig. 4G). Thus, the results suggest that the expression of endogenous miR‐140‐3p may be low in T47D. In addition, we examined the expression of miR‐140‐3p in the tumor samples by qRT‐PCR and found that the expression of miR‐140‐3p in the tumor samples was not significantly different from the expression of miR‐140‐3p in the adjacent nontumor samples (Fig. S1). The results indicated that Ki‐67 transcript isoforms with short 3′UTRs generated by alternative cleavage and polyadenylation exhibit increased stability and produce more protein due to the loss of miRNA‐mediated repression, but not because of the decreased expression of miR‐140‐3p.

## Discussion

The mechanisms to explain the increased expression of the Ki‐67 protein in cancer cells are poorly understood. Recent studies have shown that a change in 3′UTR length could alter the expression of many genes during cancer development 15, 20.

Recent studies report that messenger RNA isoforms with short 3′UTRs, generated by APA, exhibit increased stability and produce more protein by the loss of miRNA‐mediated repression. The longer 3′UTR harbors target sites for regulation by miRNAs. We hypothesized that truncations in *Ki‐67* mRNA exist in cancer cell lines and alter miRNA binding sites. Part of the *Ki‐67* oncogene upregulation observed in this study was explained by the evasion of miRNA‐mediated repression by the shorter isoform. We found that the longer *Ki‐67* 3′UTR harbors more potential binding sites than the shorter 3′UTR, such as hsa‐miR‐140‐3p. Hsa‐miR‐140‐3p can suppress breast cancer proliferation and migration, and increased expression of hsa‐miR‐140‐3p is predicted to improve breast cancer survival 28, 29.

Overall, our data identified a novel post‐transcriptional mechanism, involving APA and miRNA, that underlies the elevated expression of Ki‐67 in breast cancer. The results have shown that breast cancer cells preferentially express *Ki‐67* mRNA isoforms with short 3′UTRs, and the expression of shorter *Ki‐67* mRNAs leads to an increase in *Ki‐67* mRNA stability and translational efficiency. Our study presented here highlights the importance of APA in the regulation of *Ki‐67*.

## Author contributions

HY and RT maintained all of the cell cultures and designed the experiment. WW and MZ helped HY in performing the luciferase assays and PCR. JW helped with data collection and drafted statistical methods. HY and JH conceived the ideas of the manuscript and wrote the manuscript. JH provided funding for the experiments performed in the study. All authors read and approved the manuscript for publication.

## Supporting information


**Fig. S1.** Expression levels of miR‐140‐3p were examined by qRT‐PCR in tumor samples and their adjacent nontumor samples.
