# Supplementary figures and images for: Aberrant Ki‐67 expression through 3′UTR alternative polyadenylation in breast cancers
Source: FEBS Open Bio. 2018 Jan 26;8(3):332–8. doi: 10.1002/2211-5463.12364 (PMC5832968; doi:10.1002/2211-5463.12364)

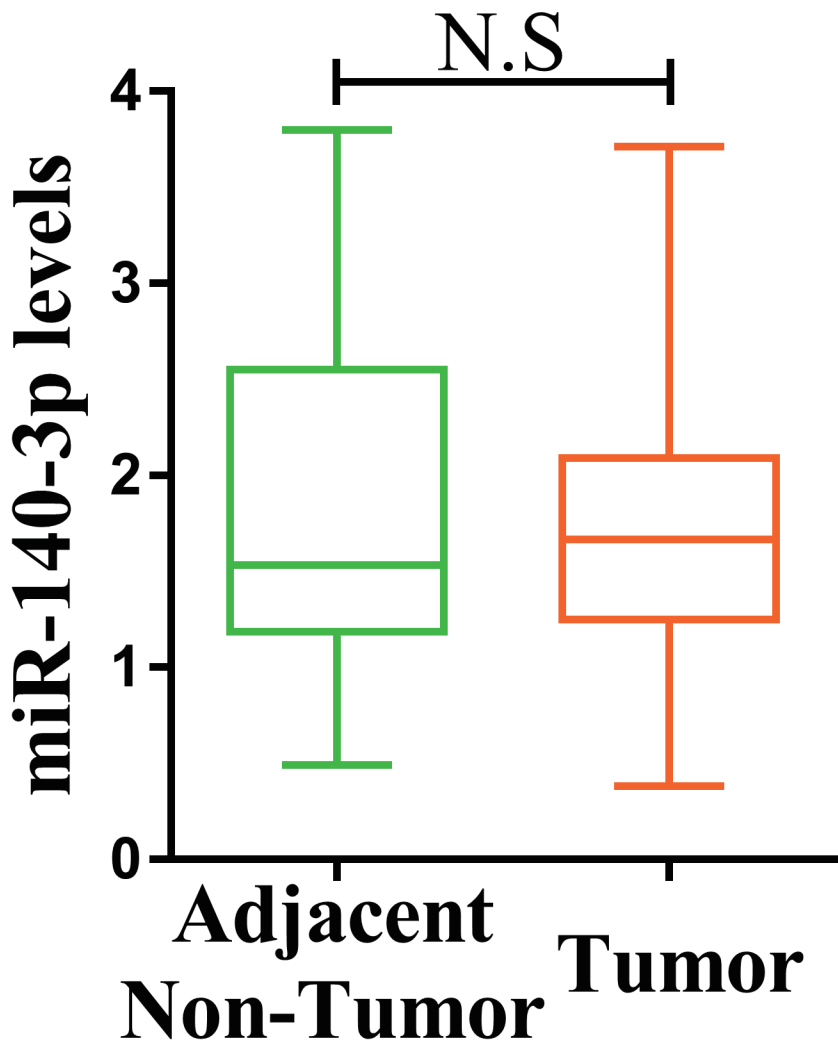

Supplement: Supplementary file 1 — Fig. S1. Expression levels of miR‐140‐3p were examined by qRT‐PCR in tumor samples and their adjacent nontumor samples. [file FEB4-8-332-s001.pdf]
